# Characteristics of adolescents with autism spectrum disorder: About 43 cases

**DOI:** 10.1192/j.eurpsy.2025.1150

**Published:** 2025-08-26

**Authors:** R. Jbir, F. Cherif, M. Bouhamed, M. Chaabene, J. Boudabous, I. Feki, F. Guermazi, J. Masmoudi

**Affiliations:** 1 psychiatry ‘A’ department; 2 Child psychiatry department, Hedi Chaker university hospital, Sfax, Tunisia

## Abstract

**Introduction:**

Autism spectrum disorder (ASD) is a neurodevelopmental disorder that appears in early childhood. The diagnosis is based on a clinical dyad: impaired social communication and restricted and repetitive behavior. From the age of 13, children with ASD are integrated into the “Erraihan” therapeutic farm.

**Objectives:**

The aim of our study is to describe the clinical profile of adolescents with ASD followed at the “Erraihan” therapeutic farm.

**Methods:**

This is a descriptive and analytical cross-sectional study of 43 parents of adolescents with ASD treated at the therapeutic farm.

The “Erraihan” therapeutic farm is a center that takes in adolescents aged over 13 with ASD.

A questionnaire using a pre-established form was administered to the parents by the same doctor to collect data relating to the adolescent.

**Results:**

Forty-three adolescents followed for autism spectrum disorder were included in our study. Their mean age was 17.79±2.29 years (min=13; max=20) with a male predominance 79.1% (n=34). Adolescents with autism were older in 48.8% of cases. They suffered from a chronic illness in 44.2% (n=19) of cases. The most frequently reported somatic antecedent was epilepsy (n=17).

Psychiatric comorbidity was found in 55.8% of adolescents. It was mainly mental retardation (n=22). Adolescent age at first consultation ranged from 1 to 4 years, with an average of 1 year 6 months. Age at diagnosis ranged from 3 to 6 years, with an average of 3 years 3 months.

Time to diagnosis ranged from 0 to 24 months, with an average of 5 months. Twenty-three adolescents (53.5%) communicated with poor speech intelligibility.

Behavioral problems were present in 72.1% of adolescents. Medication was prescribed in 62.8% of cases. The most commonly prescribed drug was risperidone (44.2%).

Sphincter autonomy was acquired in 69.8% of adolescents. Thirty-four adolescents (79.1%) had received speech therapy in childhood.

Almost half (44.2%) had entered kindergarten at an early age, and only 14% had started school and then withdrawn.

**Conclusions:**

This study sheds light on the clinical profile of adolescents with autism spectrum disorders at the “Erraihan” therapeutic farm. The results underline the importance of early and appropriate care. Although the majority of adolescents have acquired certain skills, such as sphincter autonomy and access to speech therapy, challenges remain, particularly in terms of communication and behavior. This information underlines the need for ongoing follow-up and personalized approaches to improve the quality of life of these young people within their therapeutic environment.

**Disclosure of Interest:**

None Declared

